# Metabolic engineering of fast-growing *Vibrio natriegens* for efficient pyruvate production

**DOI:** 10.1186/s12934-023-02185-0

**Published:** 2023-09-04

**Authors:** Fengli Wu, Shucai Wang, Yanfeng Peng, Yufeng Guo, Qinhong Wang

**Affiliations:** 1 Tianjin Institute of Industrial Biotechnology, Chinese Academy of Sciences, Tianjin, 300308 China; 2 National Center of Technology Innovation for Synthetic Biology, Tianjin, 300308 China; 3 College of Biotechnology, Tianjin University of Science & Technology, Tianjin, 300457 China

**Keywords:** *Vibrio natriegens*, Pyruvate, Pathway engineering, Fine-tuning gene expression, Fermentation

## Abstract

**Background:**

Pyruvate is a widely used value-added chemical which also serves as a hub of various metabolic pathways. The fastest-growing bacterium *Vibrio natriegens* is a promising chassis for synthetic biology applications with high substrate uptake rates. The aim of this study was to investigate if the high substrate uptake rates of *V. natriegens* enable pyruvate production at high productivities.

**Results:**

Two prophage gene clusters and several essential genes for the biosynthesis of byproducts were first deleted. In order to promote pyruvate accumulation, the key gene *aceE* encoding pyruvate dehydrogenase complex E1 component was down-regulated to reduce the carbon flux into the tricarboxylic acid cycle. Afterwards, the expression of *ppc* gene encoding phosphoenolpyruvate carboxylase was fine-tuned to balance the cell growth and pyruvate synthesis. The resulting strain PYR32 was able to produce 54.22 g/L pyruvate from glucose within 16 h, with a yield of 1.17 mol/mol and an average productivity of 3.39 g/L/h. In addition, this strain was also able to efficiently convert sucrose or gluconate into pyruvate at high titers.

**Conclusion:**

A novel strain of *V. natriegens* was engineered which was capable to provide higher productivity in pyruvate synthesis. This study lays the foundation for the biosynthesis of pyruvate and its derivatives in fast-growing *V. natriegens*.

**Supplementary Information:**

The online version contains supplementary material available at 10.1186/s12934-023-02185-0.

## Background

Pyruvate is an important intermediate metabolite of the glycolytic pathway. It serves as a precursor for the synthesis of many commercially valuable chemicals, such as *L*-alanine [1], *L*/*D*-lactate [2], acetoin [3], 2,3-butanediol [3], butanol [4], butyrate [5], etc. Pyruvate and its derivatives are widely utilized in food, feed, pharmaceuticals, pesticides, bioenergy, and polymer industries [6–8]. In the past two decades, microbial production of pyruvate has garnered extensive attention of researchers. A number of microorganisms, including *Escherichia coli* [9], *Corynebacterium glutamicum* [10], *Saccharomyces cerevisiae* [11], *Candida glabrata* [12, 13], and *Yarrowia lipolytica* [14] have been explored for microbial production of pyruvate.

There are three key metabolic engineering strategies to improve the pyruvate production. The first strategy is to reduce or block the carbon flux from glycolytic pathway to the tricarboxylic acid (TCA) cycle. In *E. coli*, the carbon flux can be reduced by knocking out or down-regulating the gene encoding a component of pyruvate dehydrogenase complex. Alternatively, it can be achieved by replacing the natural subunit with a less active variant [9, 15–17]. The second strategy is to block the byproduct biosynthetic pathway. Microbial cells grow rapidly under the aerobic conditions, leading to the accumulation of some byproducts, such as acetate. Knocking out the essential genes for byproduct biosynthetic pathways is helpful to improve the titers or yields of pyruvate [9, 18]. It has been reported that the deletion of *ppsA* gene encoding phosphoenolpyruvate (PEP) synthase can prevent the conversion of pyruvate to PEP in *E. coli* [9, 15]. In *S. cerevisiae*, knocking out the genes encoding pyruvate decarboxylase (PDC1, PDC5 and PDC6) resulted in blocking the pyruvate to acetaldehyde conversion, and the subsequent ethanol production through alcoholic fermentation [11, 19]. The third strategy is to maintain the intracellular NADH/NAD^+^ balance or reduce the ATP generation. Two moles of pyruvate are produced from one mole of glucose through the glycolytic pathway, which is accompanied by the generation of two moles of NADH and two moles of ATP. Introduction of water-forming NADH oxidase can provide *E. coli* cells with an alternate way to re-oxidize NADH to NAD^+^ without energy generation, resulting in an increase in the glycolytic flux [9, 18, 20]. Besides, the deletion of membrane-coupling subunit of F_0_F_1_-ATPase complex, which inactivates ATP synthesis while retaining cytoplasmic F_0_F_1_-ATPase activity, can also increase the glycolytic flux [9, 12, 20]. These metabolic engineering strategies can significantly improve pyruvate production. However, the low rates of cell growth and substrate uptake exhibited by the traditionally used microbes limit the pyruvate productivities and consume more time and energy to complete the processes of fed-batch fermentation.

*Vibrio natriegens*, a promising chassis for synthetic biology applications, is a nonpathogenic marine bacterium with ultrafast growth rate (*μ*: 4.43 1/h) and high substrate uptake rates (*qS*: 3.90 g/g/h, under aerobic conditions with glucose as substrate) [21–23]. In recent years, many strains of *V. natriegens* have been engineered by using metabolic engineering technologies to efficiently produce a variety of chemicals [21, 24–28]. For example, 2,3-butanediol productivities obtained by two engineered *V. natriegens* strains were up to 3.88 g/L/h and 3.44 g/L/h, respectively, which were 2–3 times higher than the productivity achieved by *E. coli* [25, 26]. Similarly, efficient production of 1,3-propanediol from glycerol was achieved using *V. natriegens* through systematic metabolic engineering, and the productivity reached 2.36 g/L/h [27]. In addition, the advanced synthetic biology methods and tools, such as the whole genome sequencing [29–31], genome editing [24, 32], artificial regulatory elements [33, 34] as well as metabolic flux analysis [35] are also paving the way for the industrial applications of *V. natriegens.*

The aim of this study was to investigate if the high substrate uptake rates of *V. natriegens* enable pyruvate production at high productivities. To this purpose, a series of metabolic engineering steps were employed to construct pyruvate-overproducing strains, and efficient biosynthesis of pyruvate from different substrates (glucose, sucrose and gluconate) was finally achieved.

## Results and discussion

### Deletion of prophage gene clusters to improve the cell robustness of ***V. natriegens***

There are two inducible prophage gene clusters (VPN1 and VPN2) in the genome of *V. natriegens* wild-type (WT) strain. Deletion of these two gene clusters is helpful in improving the cell robustness during cultivation process [36]. In this study, the entire sequences of VPN1 (25,777 bp) and VPN2 (39,352 bp) were aligned, analyzed, and then deleted in turn to obtain a prophage-free strain PYR02 (Table 1; Additional file 1: Fig. S1A, B). Mitomycin C (MMC), a commonly used anticancer drug, induces DNA damage via DNA alkylation to produce DNA cross-links [37]. The prophages in the genome of *V. natriegens* can be induced by MMC [36]. Under normal conditions, the growth rate of PYR02 was comparable with that of WT. However, after treatment with 1 μM MMC, only PYR02 was able to grow normally (Additional file 1: Fig. S1C). After MMC treatment for 3 to 4 h, the liquid culture of WT turned cloudy and mucous, indicating cell lysis and prophage release. The improvement of the cell robustness by knocking out the two inducible prophage gene clusters was consistent with the findings reported by a previous study [36].

**Fig. 1:**
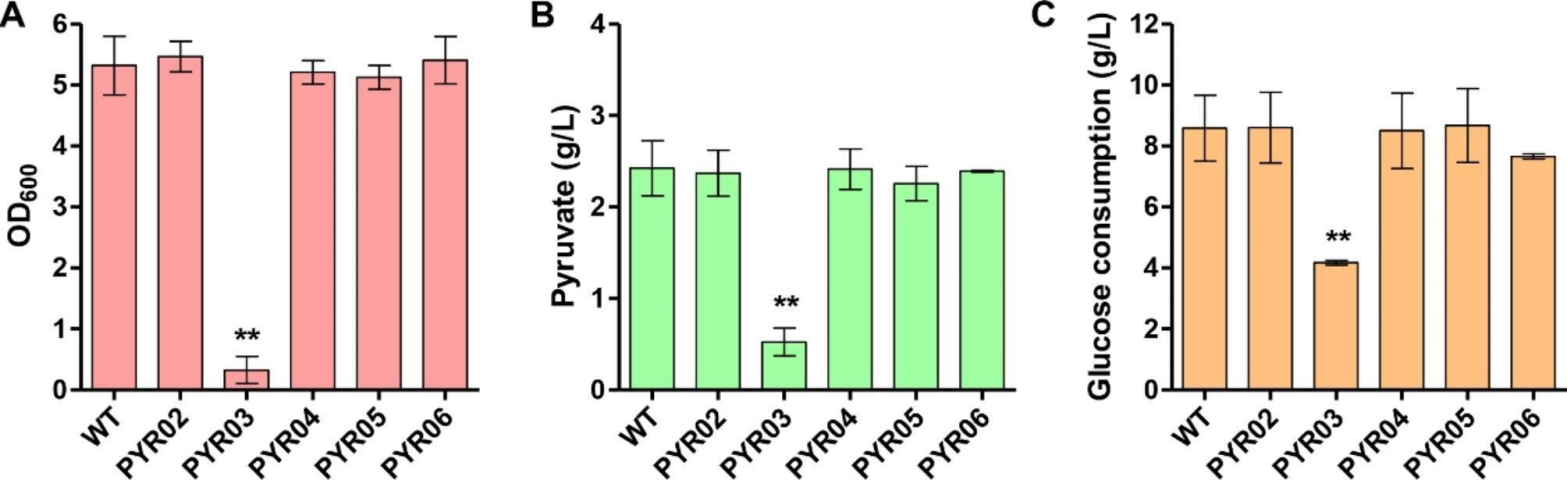
Comparison of *V. natriegens* wild-type (WT) strain to prophage-free (PYR02) and different pyruvate dehydrogenase deficient (PYR03-PYR06) strains. **A** Growth assay of WT and different engineered strains. **B** Pyruvate titers of WT and different engineered strains. **C** The amount of glucose consumed by WT and different engineered strains. The experiments were performed in shake flasks and incubated at 37 ℃ and 250 rpm for 24 h. Three independent replicates were performed. Data with error bars represent the means and standard deviations. ** *p* < 0.01

In addition, cell growth and pyruvate production in minimal medium were also examined through shaking-flask fermentation by measuring the optical density at 600 nm (OD_600_) and pyruvate titers. There were no significant differences in OD_600_, pyruvate titers, and glucose consumption between WT and PYR02 (Fig. 1A-C). In a previous study, the pyruvate titer of VPN1 and VPN2 double knockout strain was reported to be significantly higher than the parent strain [36]. This discrepancy in findings could be attributed to the differences in minimal medium composition or cultivation conditions.

### Enhancement of pyruvate accumulation by blocking catabolic pathways

Pyruvate dehydrogenase complex catalyzes the conversion of pyruvate into acetyl-CoA, which is an important precursor for the TCA cycle. Therefore, in order to accumulate pyruvate, it is necessary to reduce the activity of pyruvate dehydrogenase complex.

Four annotated genes encoding pyruvate dehydrogenase E1 component were retrieved from the genome database of *V. natriegens*. The gene numbers assigned to the four genes were BA890_11935, BA890_16565, BA890_16620 and BA890_15690, respectively [31]. Subsequently, the functions of these four genes were determined by gene knockout in prophage-free strain PYR02, and the corresponding strains were named as PYR03, PYR04, PYR05 and PYR06, respectively. During cultivation in minimal medium with glucose substrate, PYR03 grew hardly and produced a small amount of pyruvate (0.52 g/L). On the other hand, the remaining three strains showed no differences in OD_600_, pyruvate titers and glucose consumption, as compared to PYR02 (Fig. 1A-C), indicating that the gene numbered as BA890_11935 encoded a functional pyruvate dehydrogenase E1 component in *V. natriegens* cells and was named as *aceE*.

**Fig. 2:**
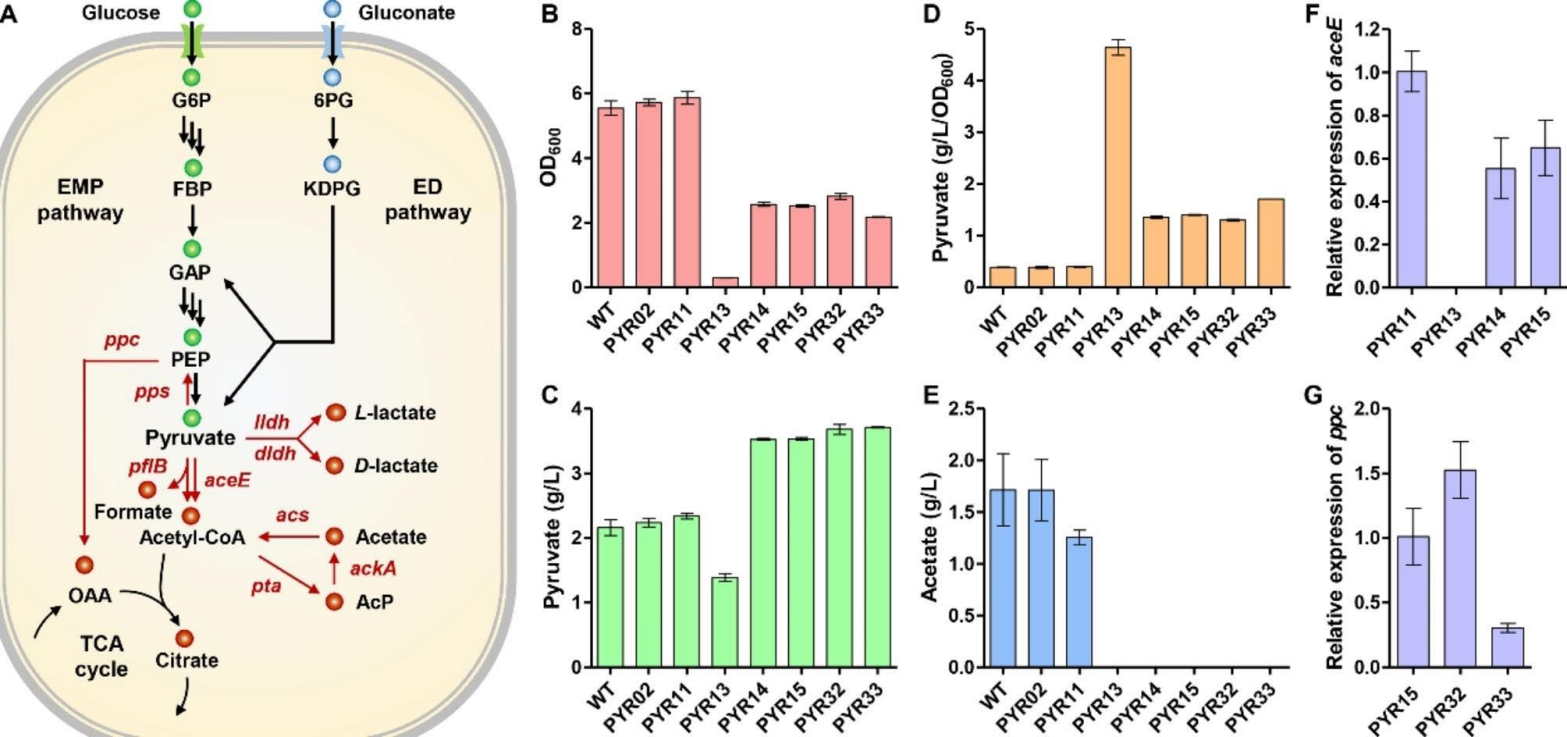
Improvement of pyruvate production by pathway engineering. **A** Schematic diagram of metabolic engineering strategies. The genes marked in red color are essential genes for the biosynthesis of byproducts. **B** Growth assay of PYR02 and other engineered strains. **C** Pyruvate titers of PYR02 and other engineered strains. **D** Pyruvate titers of per OD_600_ cells for PYR02 and other engineered strains. **E** Acetate titers of PYR02 and other engineered strains. **F** qRT-PCR analysis of *aceE* transcription level. **G** qRT-PCR analysis of *ppc* transcription level. The experiments were performed in shake flasks and incubated at 37 ℃ and 250 rpm for 24 h. Three independent replicates were performed. Data with error bars represent the means and standard deviations

Due to the serious impact of *aceE* knockout on cell growth, the genes annotated as encoding pyruvate formate lyase (PflB), *L*-lactate dehydrogenase (Lldh), *D*-lactate dehydrogenase (Dldh), PEP synthase (Pps1 and Pps2) were first deleted in PYR02 to reduce the accumulation of byproducts, such as formate, *L*-lactate, *D*-lactate, and PEP (Fig. 2A). The resulting strain was named as PYR11. Compared with PYR02, the OD_600_ and pyruvate titer of PYR11 increased by 2.35% and 4.51%, respectively, while the acetate byproduct production decreased by 26.63% (Fig. 2B, C and E). These results indicate that blocking the byproduct biosynthetic pathways is helpful in promoting the pyruvate biosynthesis.

Next, in order to reduce the carbon flux entering the TCA cycle, the effects of gene deletion and down-regulation of *aceE* were examined for strain PYR11. The artificial regulatory part P2, including the promoter region and ribosome binding site (RBS) element, is a constitutive promoter with low expression activity [34]. Then, the regulatory part P2 and two rare start codons (GTG and TTG) were used to down-regulate the expression of *aceE*, generating strains PYR14 and PYR15, respectively. Similar to PYR03, the *aceE* knockout strain PYR13 grew hardly in minimal medium, but still produced 1.39 g/L of pyruvate with a titer of 4.64 g/L per OD_600_ cells (Fig. 2B-D). Compared to PYR11, OD_600_ of PYR14 and PYR15 decreased by 56.07% and 56.93%, respectively (Fig. 2B). However, the pyruvate titers of PYR14 and PYR15 increased to 3.53 g/L and 3.54 g/L, respectively, which were approximately 50% higher than that of PYR11 (Fig. 2C). Similarly, the pyruvate titers of per OD_600_ cells of PYR14 and PYR15 reached 1.36 g/L and 1.40 g/L, respectively, which were approximately 2.5-fold higher than that of PYR11 (Fig. 2D). In addition, no detectable acetate accumulation was observed in the broths of PYR13, PYR14 and PYR15 (Fig. 2E). After deletion or down-regulation of *aceE*, the intracellular pyruvate cannot be extensively converted into acetyl-CoA, which is the direct precursor of acetate biosynthesis through the phosphate acetyltransferase-acetate kinase (Pta-AckA) pathway (Fig. 2A). Moreover, genome sequence analysis revealed that there was no annotated gene encoding pyruvate oxidase (PoxB), which catalyzes the acetate biosynthesis from pyruvate, in the genome of *V. natriegens* [31]. These facts clearly explain the absence of acetate accumulation in the fermentation broth.

The transcription level of *aceE* was further analyzed by quantitative real-time polymerase chain reaction (qRT-PCR). As shown in Fig. 2F, the transcription levels of *aceE* in PYR14 and PYR15 decreased by approximately 40%, indicating that the regulatory part P2 was weaker than the native promoter. No transcripts were observed in the *aceE* knockout strain PYR13. ATG is a common start codon; however, GTG and more rarely TTG are also employed by some genes. Use of the rare start codon can reduce the translational initiation efficiency, thereby down-regulating the expression level of the target gene, which is a commonly used strategy in metabolic engineering research [38–40]. Since the rare start codons also exist in the genome of *V. natriegens* [31], the enzymic content or activity of pyruvate dehydrogenase in PYR14 and PYR15 would further reduce at the translation level. These results indicate that the modulation of *aceE* by using the combination of artificial regulatory part and rare start codons may lead to more reasonable distribution of intracellular carbon flux, thereby maintaining the balance between cell growth and pyruvate biosynthesis.

### Regulating the expression of ***ppc*** to improve cell growth and pyruvate production

PEP carboxylase encoded by *ppc* gene catalyzes the conversion of PEP to oxaloacetate, which is an intermediate metabolite in the TCA cycle. It has been reported that approximately 27% of the carbon flux enters the TCA cycle *via* oxaloacetate anaplerotic pathway catalyzed by PEP carboxylase in *V. natriegens* [35]. Therefore, regulating the expression of *ppc* was done in PYR15 by using the regulatory part P2 and two different start codons (ATG and TTG) and the resulting strains were named as PYR32 and PYR33, respectively. The three strains were examined to determine the effect of *ppc* expression on pyruvate production. Compared to PYR15 (OD_600_: 2.52), the OD_600_ increased to 2.82 for PYR32 and decreased to 2.18 for PYR33 (Fig. 2B). Pyruvate titers of PYR32 and PYR33 increased to 3.68 g/L and 3.71 g/L, respectively, which were 4.29% and 5.08% higher than PYR15 (Fig. 2C). Due to the higher pyruvate titer and lower OD_600_, the pyruvate titer per OD_600_ cells for PYR33 increased by 22.11% (Fig. 2D). Surprisingly, the transcription levels of *ppc* in these two strains were not consistent, even though both PYR32 and PYR33 employed the same regulatory part P2. Compared with PYR15, the transcription level of *ppc* in PYR32 was up-regulated by approximately 1.5 times. On the contrary, *ppc* transcription was observed to be down-regulated in PYR33 by more than 3 times (Fig. 2G). This inconsistency may be attributed to the rare start codon TTG of *ppc*, which may affect the transcriptional activity of the regulatory part P2 or the stability of mRNA. These results suggest that regulating the expression of *ppc* in *V. natriegens* may affect the cell growth and pyruvate biosynthesis by redirecting the carbon flux entering the TCA cycle.

### High-level production of pyruvate by using fed-batch fermentation

**Fig. 3:**
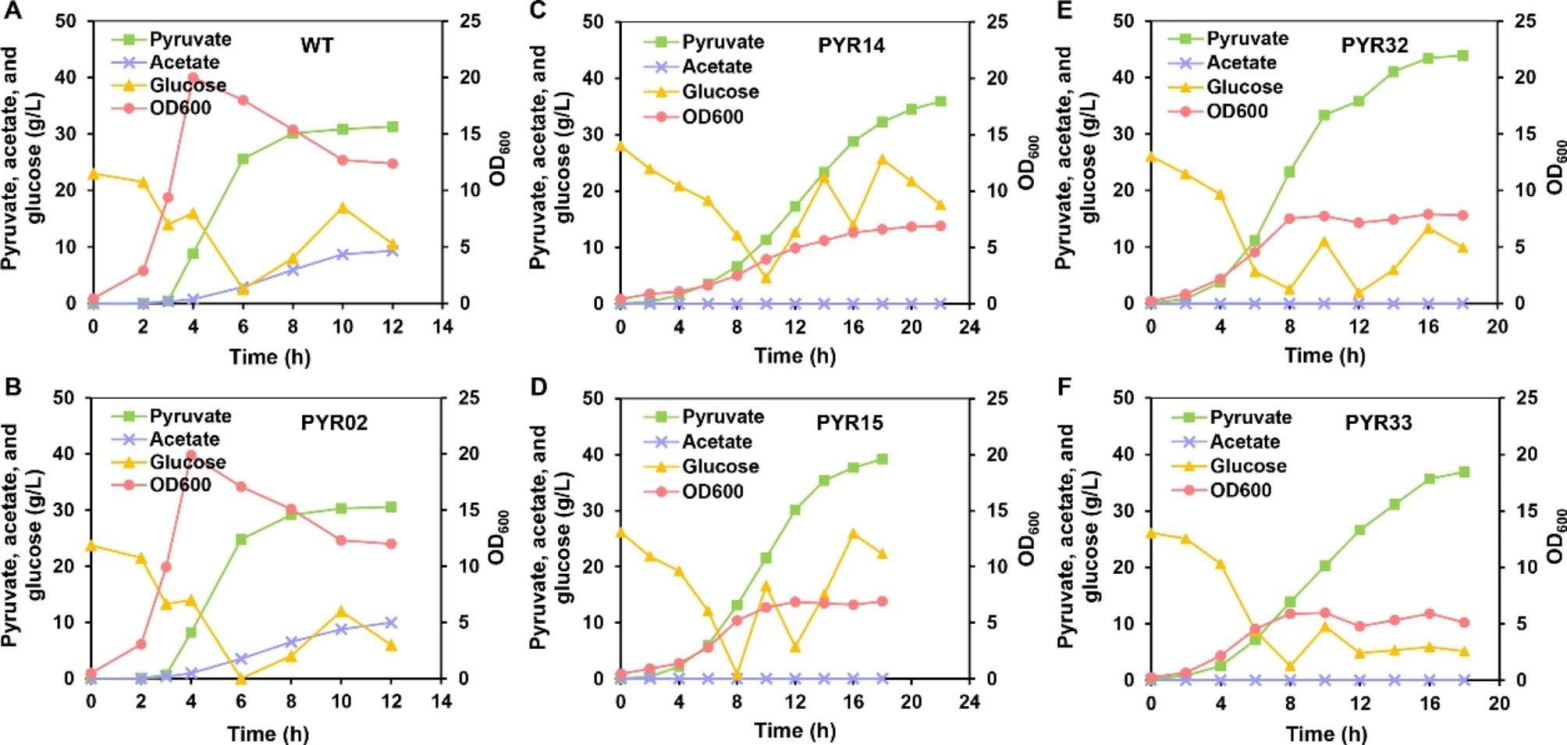
Improvement of pyruvate production by using fed-batch fermentation. **A**-**F** Fermentation results of the engineered strains (**A** WT, **B** PYR02, **C** PYR14, **D** PYR15, **E** PYR32, and **F** PYR33). The processes were conducted at 37 ℃ and glucose was used as carbon source. Fed-batch fermentation for each strain was performed two times, and the result of one representative fed-batch culture have been presented here

High-level production of pyruvate by the engineered strains was studied through fed-batch fermentation which was conducted in 5-L fermenters. The results revealed that the growth rates of WT and PYR02 were comparable and extremely fast with almost same pyruvate titers (31.31 g/L vs. 30.58 g/L), yields (0.62 mol/mol vs. 0.61 mol/mol) and productivities (2.61 g/L/h vs. 2.55 g/L/h) (Fig. 3A, B; Additional file 1: Table S1). Moreover, both strains produced a large amount of acetate as byproduct, and the titers reached to 9.30 g/L and 9.97 g/L, respectively. These results were consistent with the findings of shaking-flask fermentation.

After blocking pyruvate catabolic pathways, the pyruvate titers of PYR14 and PYR15 increased to 35.88 g/L and 39.19 g/L, respectively, which were 17.33% and 28.16% higher than that of PYR02 (Fig. 3C, D). The OD_600_ of PYR14 and PYR15 was only 6.90, and no accumulation of acetate was observed. Therefore, the yields of these two strains were found to be 104.92% and 106.56% higher and reached to 1.25 mol/mol and 1.26 mol/mol on glucose, respectively (Additional file 1: Table S1). However, the growth rates of PYR14 and PYR15 strains were slower than the PYR02, resulting in decreased productivities of 1.63 g/L/h and 2.18 g/L/h, respectively (Additional file 1: Table S1). In addition, neither of these two strains produced acetate due to the down-regulation of *aceE*.

After regulating the expression of *ppc* by using the regulatory part P2 and the common start codon ATG, the pyruvate titer for PYR32 further increased to 43.95 g/L, with a productivity of 2.44 g/L/h (Fig. 3E; Additional file 1: Table S1). The *ppc* transcription level in PYR32 was found to be approximately 0.5-fold higher than the transcription level in PYR15, which might be the reason of increased OD_600_ value (7.80) for PYR32 (Figs. 2G and 3E). The increase in OD_600_ indicated to higher glucose consumption by the strain for cell growth, resulting in the decreased yield of 1.18 mol/mol. On the other hand, regulating the expression of *ppc* in PYR33 by using the regulatory part P2 and rare start codon TTG resulted in pyruvate titer of 36.91 g/L and productivity of 2.05 g/L/h (Fig. 3F; Additional file 1: Table S1). The *ppc* transcription level in PYR33 was found to be approximately one-third of the transcription level in PYR15, causing a decrease in final OD_600_ to 5.12 (Figs. 2G and 3F). The decrease in OD_600_ was accompanied by decrease in glucose consumption and increase in yield (1.30 mol/mol) (Additional file 1: Table S1). Compared to the results of shaking-flask fermentation, the pyruvate titers for these strains increased by approximately 10 times in the fed-batch fermentation.

**Fig. 4:**
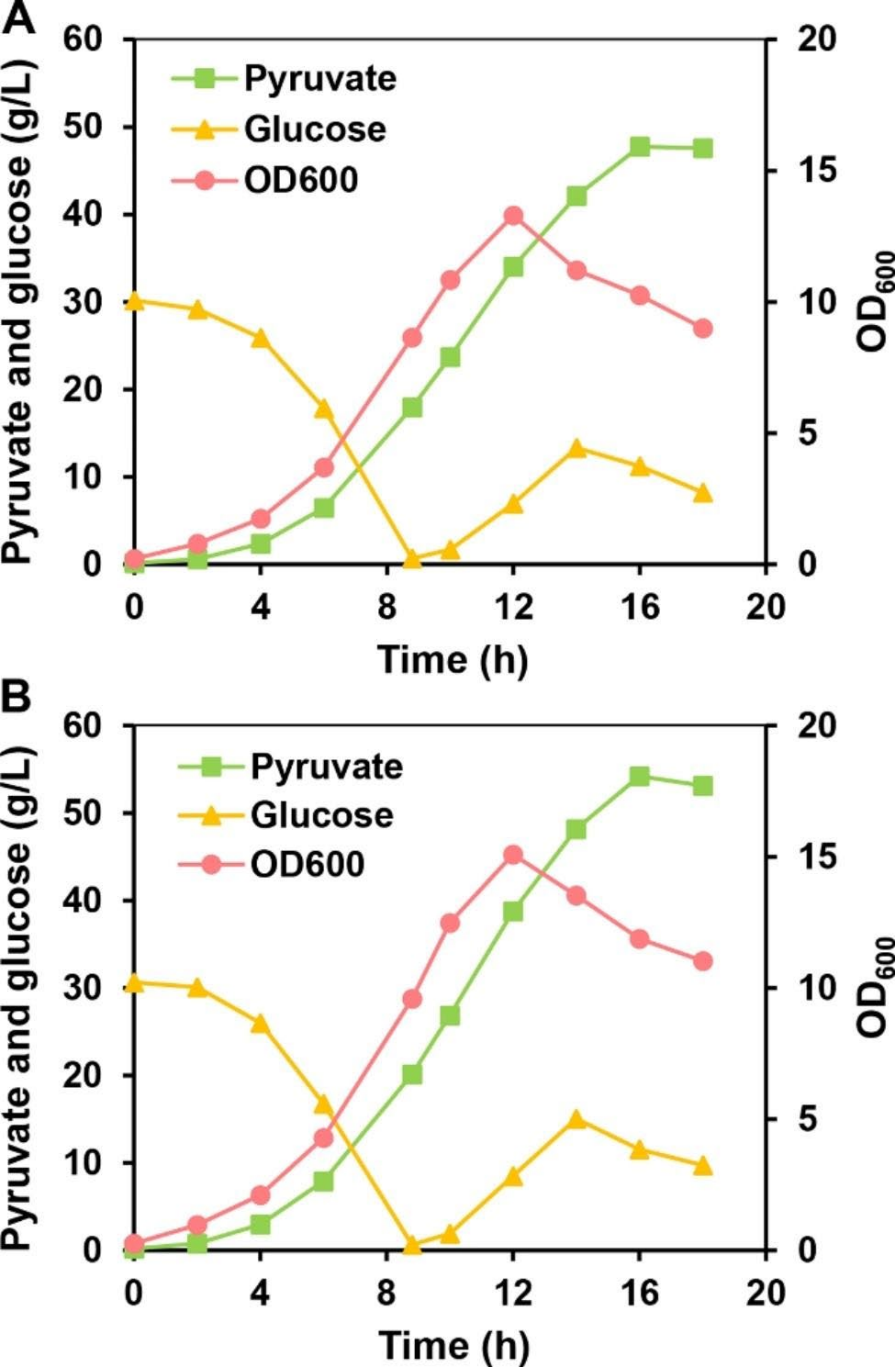
Improvement of pyruvate production by the optimization of cultivation temperature. Fermentation results of PYR15 (**A**) and PYR32 (**B**). The processes were conducted at 30 ℃ and glucose was used as carbon source. Fed-batch fermentation for each strain was performed two times, and the result of one representative fed-batch culture have been presented here

Cultivation temperature is an important factor that affects the cell growth and production of desired chemical. When the temperature decreased from 37 ℃ to 30 ℃ during fed-batch fermentation, the biomass of *V. natriegens* increased by 62%, while the concentrations of byproducts such as acetate and lactate significantly decreased [41]. Therefore, PYR15 and PYR32 were selected to determine the optimum cultivation temperature in fed-batch fermentation. When cultivated at 30 ℃, the pyruvate titer of PYR15 increased by 21.89% and reached 47.77 g/L, with a yield of 1.16 mol/mol and a productivity of 2.99 g/L/h (Fig. 4A; Additional file 1: Table S1). Similarly, the pyruvate titer of PYR32 also increased by 23.37% and reached 54.22 g/L, with a yield of 1.17 mol/mol and a higher productivity (3.39 g/L/h) (Fig. 4B; Additional file 1: Table S1). The maximum OD_600_ for PYR15 and PYR32 at 30℃ (13.29 and 15.09) were approximately 1.9-fold of the OD_600_ values at 37℃ (6.90 and 7.90). Interestingly, the time needed for fed-batch fermentation was shortened to 16 h at 30 ℃ (16 h vs. 18 h) (Figs. 3D and E and 4A and B).

Overall, this study suggests that the engineered *V. natriegens* strains were able to carry out biosynthesis of pyruvate from glucose at high productivities. As shown in Table 1, the productivity of pyruvate exhibited by *V. natriegens* was the highest among the studied microorganisms, despite the uncompetitive pyruvate titers.

**Table 1:** Production of pyruvate from glucose by different microorganisms

Strain	Titer (g/L)	Time (h)	Productivity (g/L/h)	Yield (mol/mol)	References
*E. coli*	62	36	1.72	1.11	[42]
*E. coli*	90	44	2.05	1.39	[9]
*E. coli*	93	46	2.02	1.45	[18]
*C. glutamicum*	45.1	105	0.43	0.97	[10]
*S. cerevisiae*	135	100	1.35	1.10	[11]
*S. cerevisiae*	24.7	96	0.26	0.63	[19]
*C. glabrata*	49.8	40	1.25	1.06	[12]
*C. glabrata*	94.3	82	1.15	1.30	[43]
*C. glabrata*	53.1	72	0.74	ns	[44]
*V. natriegens*	54.2	16	3.39	1.17	This study

ns, not specified

### Production of pyruvate with different carbon sources

**Fig. 5:**
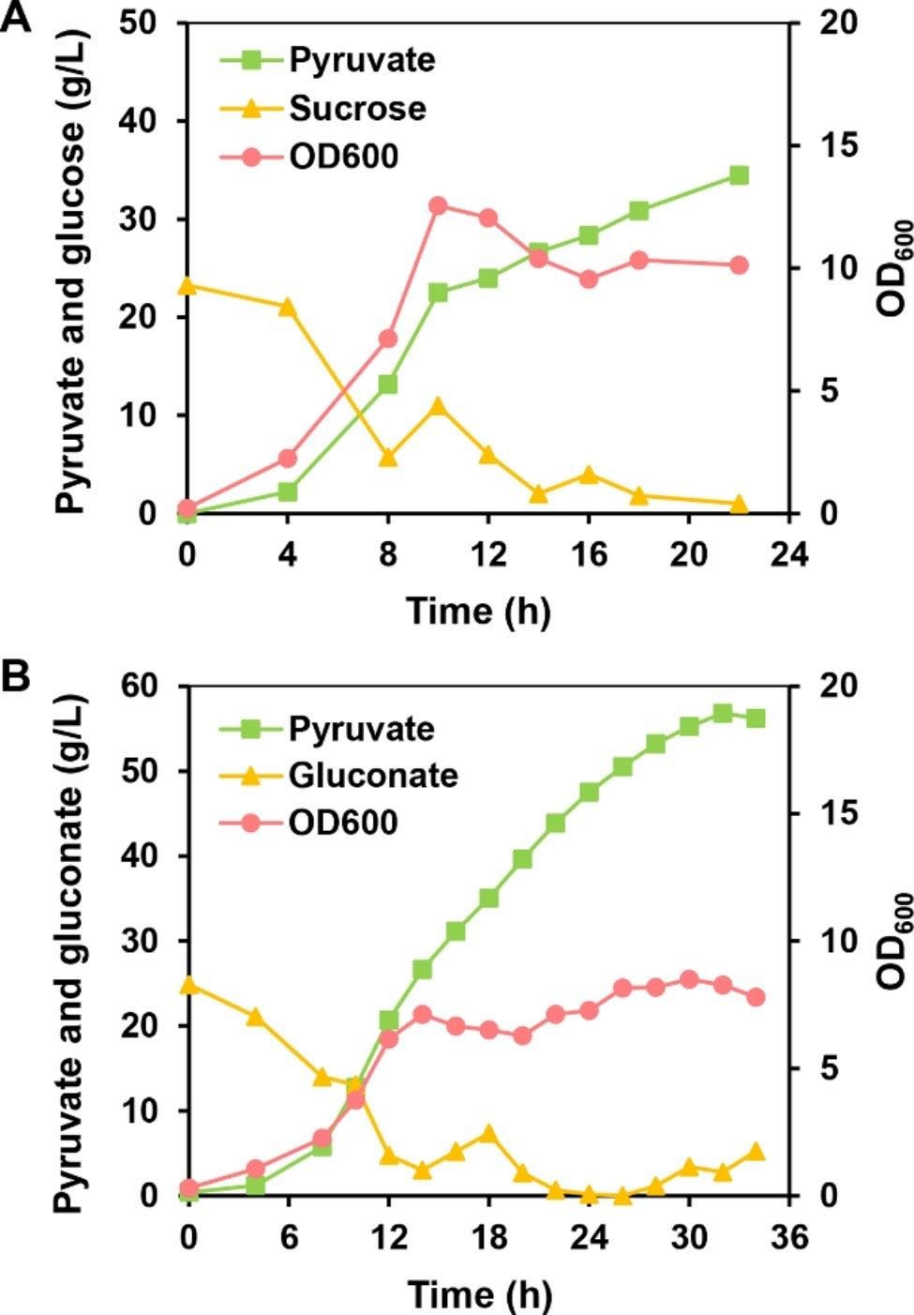
High-level production of pyruvate by PYR32 using different carbon sources. **A** Biosynthesis of pyruvate from sucrose. **B** Biosynthesis of pyruvate from gluconate. Fed-batch fermentation was performed two times at 30 ℃, and the result of one representative fed-batch culture have been presented here

*V. natriegens* is a robust microorganism and exhibit ultrafast aerobic growth with a variety of carbon sources, such as sucrose (*μ* = 1.79 1/h), glucose (*μ* = 1.68 1/h), fructose (*μ* = 1.51 1/h), gluconate (*μ* = 1.51 1/h), and *N*-acetylglucosamine (*μ* = 1.74 1/h) [21]. In this study, two industrially relevant carbon sources, sucrose and gluconate, were used as substrates for the fed-batch fermentation. When sucrose was used as carbon source, PYR32 produced 34.49 g/L of pyruvate in 22 h, with a yield of 1.32 mol/mol and a productivity of 1.57 g/L/h (Fig. 5A; Additional file 1: Table S1). However, when gluconate was used as carbon source, a pyruvate titer of 56.83 g/L was achieved in 32 h, with a yield of 1.31 mol/mol and a productivity of 1.78 g/L/h (Fig. 5B; Additional file 1: Table S1). It has been reported that in minimal medium with gluconate carbon source, intracellular gluconokinase and enzymes involved in the Entner-Doudoroff (ED) pathway were induced in *V. natriegens*, dissimilating approximately 80% of gluconate *via* the ED pathway [45]. These results indicate that the engineered *V. natriegens* is also able to efficiently convert sucrose or gluconate into pyruvate at high titers.

## Conclusion

With ultrafast growth rate and high substrate uptake rate, *V. natriegens* has emerged as a promising chassis in synthetic biology to produce value-added chemicals. In this study, a series of metabolic engineering strategies (i.e., deletion of two inducible prophage gene clusters and other essential genes; and fine-tuning of *aceE* and *ppc* expression) were employed to construct a pyruvate-overproducing strain. The engineered strain PYR32 was able to produce 54.22 g/L of pyruvate from glucose within 16 h, with a yield of 1.17 mol/mol and an average productivity of 3.39 g/L/h (Fig. 4B; Additional file 1: Table S1). Additionally, this strain was also capable of efficient pyruvate production from other industrially relevant carbon sources such as sucrose or gluconate. This study lays the foundation for the biosynthesis of pyruvate and its derivatives by using *V. natriegens*. However, further studies are needed to improve the cell viability of *V. natriegens*, especially after cells enter the stationary phase during the fermentation process. In addition, as a marine bacterium, *V. natriegens* possesses high salt tolerance. Therefore, *V. natriegens* can be used in a non-sterilized continuous fermentation process directly consuming seawater, which can further reduce the production cost of pyruvate.

## Materials and methods

### Strains and genetic manipulations

*V. natriegens* CICC 10,908 (= ATCC 14,048) was used in this study. Seamless genetic manipulations, including gene knockout and regulation, were conducted according to the previously described methods [46]. WT and engineered strains have been listed in Table 2. Primers used for strain construction have been listed in Additional file 1: Table S2.

**Table 2:** Strains used in this study

Strain	Relevant genotype	Source
*V. natriegens* CICC 10,908	Referred here as the wild-type strain	China Center of Industrial Culture Collection
PYR02	Δ*VPN1*, Δ*VPN2*	This study
PYR03	Δ*VPN1*, Δ*VPN2*, Δ*aceE*	This study
PYR04	Δ*VPN1*, Δ*VPN2*, Δ*ace1*	This study
PYR05	Δ*VPN1*, Δ*VPN2*, Δ*ace2*	This study
PYR06	Δ*VPN1*, Δ*VPN2*, Δ*ace3*	This study
PYR11	Δ*VPN1*, Δ*VPN2*, Δ*pflB*, Δ*lldh*, Δ*dldh*, Δ*pps1*, Δ*pps2*	This study
PYR13	Δ*VPN1*, Δ*VPN2*, Δ*pflB*, Δ*lldh*, Δ*dldh*, Δ*pps1*, Δ*pps2*, Δ*aceE*	This study
PYR14	Δ*VPN1*, Δ*VPN2*, Δ*pflB*, Δ*lldh*, Δ*dldh*, Δ*pps1*, Δ*pps2*, P2-*aceE*^GTG^	This study
PYR15	Δ*VPN1*, Δ*VPN2*, Δ*pflB*, Δ*lldh*, Δ*dldh*, Δ*pps1*, Δ*pps2*, P2-*aceE*^TTG^	This study
PYR32	Δ*VPN1*, Δ*VPN2*, Δ*pflB*, Δ*lldh*, Δ*dldh*, Δ*pps1*, Δ*pps2*, P2-*aceE*^TTG^, P2-*ppc*^ATG^	This study
PYR33	Δ*VPN1*, Δ*VPN2*, Δ*pflB*, Δ*lldh*, Δ*dldh*, Δ*pps1*, Δ*pps2*, P2-*aceE*^TTG^, P2-*ppc*^TTG^	This study

^ATG/GTG/TTG^, representing different start codons

### Media and growth conditions

During strain construction, cultures were routinely grown in LB3 medium at 30 ℃. LB3 medium consisted of 10 g/L tryptone, 5 g/L yeast extract, and 30 g/L NaCl [32]. Spectinomycin (200 μg/mL) was added into the LB3 medium to maintain the stability of the genome editing plasmid (pMB1-tfoX) [46].

For shaking-flask experiments, single colonies were inoculated into 5 mL of LB3 medium and cultured overnight in shaking incubator at 30 ℃ and 250 rpm. The overnight seed cultures were then inoculated into a 100-mL shake flask containing 15 mL of shaking-flask (SF) medium at a ratio of 1:100 and incubated at 37 ℃ and 250 rpm for 24 h. The SF medium contained 7.5 g/L K_2_HPO_4_·3H_2_O, 2 g/L MgSO_4_·7H_2_O, 1.6 g/L (NH_4_)_2_SO_4_, 2 g/L citric acid monohydrate, 0.075 g/L FeSO_4_·7H_2_O, 20 g/L NaCl, 20 g/L glucose, 20.9 g/L 3-(N-morpholino)propanesulfonic acid (MOPS), and 1 mL trace element solution. The trace element solution consisted of 4.5 g/L MnSO_4_·H_2_O, 20 g/L Na_2_SO_4_, 6.4 g/L ZnSO_4_·7H_2_O, 4 g/L CoCl_2_·6H_2_O, and 0.6 g/L CuSO_4_·5H_2_O.

For fed-batch fermentation, the seed was prepared in the same way as shaking-flask experiment. The seed culture was then inoculated into a 1-L shake flask containing 200 mL of LB3 medium at a ratio of 1:1000 and incubated at 30 °C and 250 rpm for 10–12 h. Subsequently, the culture was transferred into a 5-L fermenter (Bxbio, Shanghai, China) containing 2.3 L of fed-batch (FB) medium and cultivated at the studied temperature (30 or 37 °C). The FB medium contained 7.5 g/L K_2_HPO_4_·3H_2_O, 2 g/L MgSO_4_·7H_2_O, 1.6 g/L (NH_4_)_2_SO_4_, 2 g/L citric acid monohydrate, 0.075 g/L FeSO_4_·7H_2_O, 20 g/L NaCl, and 1 mL trace element solution. The composition of trace element solution was same as the one used for SF medium. NaCl was not added in the medium when the fermentation process was fed with sodium gluconate. The feeding medium contained 600 g/L glucose or 550 g/L sucrose or 500 g/L sodium gluconate, according to the design of experiment. The initial substrate concentration was kept at approximately 25–30 g/L. After the initial substrate was exhausted, the DO-stat feeding strategy was employed to supply feeding medium into the bioreactor and keep substrate concentration approximately 5–10 g/L. The airflow was kept at 1.0 vvm, and the agitation was changed to maintain the dissolved oxygen (DO) concentration above 30% saturation. The pH was maintained at 7.0 by automatic addition of 25% (w/v) NH_3_·H_2_O (for glucose or sucrose carbon source) or 8 M NaOH solution (for sodium gluconate carbon source). The fed-batch fermentation process was ended when the pyruvate titer dose not increase.

### qRT-PCR analysis

The total RNA was extracted from chemostat samples (10^9^ cells) using the RNAprep Pure Cell/Bacteria Kit (TIANGEN, Beijing, China) according to the manufacturer´s instructions. First-strand cDNA synthesis was carried out from 800 ng of total RNA using the PrimeScript RT reagent Kit (Takara Biomedical Technology, Beijing, China) as per the manufacturer´s protocol. TB Green Premix Ex Taq II (Tli RNaseH Plus) Kit (Takara Biomedical Technology, Beijing, China) was used for qRT-PCR conducted in a LightCycler 96 (Roche, Basel, Switzerland). The transcription levels were calculated using the 2^−ΔΔCT^ method [47] and normalized against the 16 S ribosomal RNA (rRNA) gene as an internal control. Primers used for qRT-PCR have been listed in Additional file 1: Table S2.

### Analytical methods

Cell growth was monitored by measuring the OD_600_ using a SP-723 spectrophotometer (Shanghai Spectrum Instruments Ltd., Shanghai, China). The concentrations of pyruvate, acetate and residual glucose, sucrose or gluconate were determined by a 1260 high-performance liquid chromatography (HPLC) (Agilent Technologies, Palo Alto, CA, USA) equipped with a RezexTM RFQ-Fast Acid H+ (8%) column (100 × 7.8 mm) (Phenomenex, Torrance, CA, USA). The culture samples were centrifuged at 12,000 rpm for 10 min to remove cells, and the supernatant was filtered through a 0.22 μm polyethersulfone (PES) membrane filter. The injection volume was 5 μL. The analysis was performed at 55 ℃ with a mobile phase of 5 mM H_2_SO_4_, at a flow rate of 0.6 mL/min. The diode array detector (DAD) was used to monitor the signals for pyruvate and acetate at 210 nm. Glucose, sucrose, and gluconate were measured by using refractive index detector (RID). Data processing and statistical analysis were conducted using EXCEL software (Microsoft, Redmond, Washington State, USA).

### Electronic supplementary material

Below is the link to the electronic supplementary material.

**Additional file 1: Figure S1**. Analysis the functions of two inducible prophages in the genome of *V. natriegens* wild-type (WT) strain. **Table S1**. Fed-batch fermentation parameters for WT and engineered strains. **Table S2**. Primers used in this study.

## Data Availability

All data generated or analyzed in this study are included in this article and its supplementary information files.
