# Supplementary material for: Metabolic engineering of fast-growing Vibrio natriegens for efficient pyruvate production
Source: Microb Cell Fact. 2023 Sep 4;22:172. doi: 10.1186/s12934-023-02185-0 (PMC10476420; doi:10.1186/s12934-023-02185-0)
Supplement: Supplementary file 1 — Additional file 1: Figure S1. Analysis the functions of two inducible prophages in the genome of V. natriegens wild-type (WT) strain. Table S1. Fed-batch fermentation parameters for WT and engineered strains. Table S2. Primers used in this study. [file 12934_2023_2185_MOESM1_ESM.docx]

**Supplementary materials**

**Metabolic engineering of fast-growing** ***Vibrio natriegens* for efficient** **pyruvate production**

Fengli Wu^1,2,*^, Shucai Wang^1,2,3^, Yanfeng Peng^1,2^, Yufeng Guo^1,2^, Qinhong Wang^1,2,*^

^1^ Tianjin Institute of Industrial Biotechnology, Chinese Academy of Sciences, Tianjin 300308, China

^2^ National Center of Technology Innovation for Synthetic Biology, Tianjin 300308, China

^3^ College of Biotechnology, Tianjin University of Science & Technology, Tianjin 300457, China

^*^ Correspondence: wu_fl@tib.cas.cn; wang_qh@tib.cas.cn

Mailing address: Tianjin Institute of Industrial Biotechnology, Chinese Academy of Sciences, 32 XiQiDao, Tianjin Airport Economic Area, Tianjin 300308, P. R. China

Phone: +86-022-84861950

**Supplementary Figure**

**Fig. S1** Analysis the functions of two inducible prophages in the genome of *V. natriegens* wild-type (WT) strain. **A** and **B** Schematic diagram of the prophage gene clusters VPN1 and VPN2. Both clusters are located on chromosome 1 (RefSeq accession number CP016345.1). **C** Growth curves of WT and PYR02 cultured at 30 ℃ and 250 rpm in BHIN medium (37 g/L brain heart infusion and 15 g/L NaCl) with or without 1 μM MMC. Three independent replicates were performed. Data with error bars represent the means and standard deviations.

**Supplementary Tables**

Table S1 Fed-batch fermentation parameters for WT and engineered strains.

| **Strain** | **Temperature**  **(℃)** | **Substrate** | **Substrate consumption**  **(g)** | **Time**  **(h)** | **Pyruvate titer**  **(g/L)** | **Acetate titer**  **(g/L)** | **Volume**  **(L)** | **Yield**  **(mol/mol)** | **Productivity**  **(g/L/h)** |
| --- | --- | --- | --- | --- | --- | --- | --- | --- | --- |
| WT | 37 | Glucose | 308 | 12 | 31.31 | 9.30 | 3.00 | 0.62 | 2.61 |
| PYR02 | 37 | Glucose | 311 | 12 | 30.58 | 9.97 | 3.01 | 0.61 | 2.55 |
| PYR14 | 37 | Glucose | 164 | 22 | 35.88 | 0 | 2.80 | 1.25 | 1.63 |
| PYR15 | 37 | Glucose | 179 | 18 | 39.19 | 0 | 2.81 | 1.26 | 2.18 |
| PYR32 | 37 | Glucose | 213 | 18 | 43.95 | 0 | 2.80 | 1.18 | 2.44 |
| PYR33 | 37 | Glucose | 160 | 18 | 36.91 | 0 | 2.75 | 1.30 | 2.05 |
| PYR15 | 30 | Glucose | 238 | 16 | 47.77 | 0 | 2.82 | 1.16 | 2.99 |
| PYR32 | 30 | Glucose | 261 | 16 | 54.22 | 0 | 2.75 | 1.17 | 3.39 |
| PYR32 | 30 | Sucrose | 295 | 22 | 34.49 | 0 | 2.90 | 1.32 | 1.57 |
| PYR32 | 30 | Gluconate | 288 | 32 | 56.83 | 0 | 2.97 | 1.31 | 1.78 |

Table S2 Primers used in this study.

| **Primer name** | **Primer sequence (5′ to 3′)** | **Description** |
| --- | --- | --- |
| CS-BamHI-F | AGTGGGATCCGTGACGGAAGATCACTTC | Amplification of *cat*-*sacB* gene cassette |
| CS-XhoI-R | GAGGCTCGAGATCAAAGGGAAAACTGTCC |  |
| VPN1-D-1F | AGAATTGAACTTGGCTCTGTCAC | Deletion of prophage gene cluster VPN1 |
| VPN1-D-1R | GAAAGAGGAGCAAGTGGTGT |  |
| VPN1-BamHI-R | ACGTGGATCCGAAAGAGGAGCAAGTGGTGT |  |
| VPN1-XhoI-F | CTCACTCGAGCCCCTTCAACTTATCAATCG |  |
| VPN1-D-2F | ACACCACTTGCTCCTCTTTCCCCCTTCAACTTATCAATCG |  |
| VPN1-D-2R | AACGCAATACCGAAGCCACC |  |
| VPN2-D-1F | GATCGAGGCAATCGCAAGTTTAACC | Deletion of prophage gene cluster VPN2 |
| VPN2-D-1R | TAACTCGCAACGAAAGATG |  |
| VPN2-BamHI-R | ACGTGGATCCTAACTCGCAACGAAAGATG |  |
| VPN2-XhoI-F | CTCACTCGAGCATCGGCGAGCCTTTAGTT |  |
| VPN2-D-2F | CATCTTTCGTTGCGAGTTACATCGGCGAGCCTTTAGTT |  |
| VPN2-D-2R | GCAACAGGCACAATCCAGC |  |
| aceEF-D-1F | GCTGGATTTACTGGAAACG | Deletion and down-regulation of *aceE* |
| aceEF-D-1R | GGCGATCTATCCTTCTGTTGG |  |
| aceEF-W-1R | GTCACACTTTTTTCAAATAAGCTTGGCGATCTATCCTTCTGTTGG |  |
| aceEF-BamHI-R | TCGTGGATCCGGCGATCTATCCTTCTGTTGG |  |
| aceEF-XhoI-F | GACGCTCGAGTAGAAGTTAGATGTATGAGAGGC |  |
| aceEF-D-2F | CAACAGAAGGATAGATCGCCTAGAAGTTAGATGTATGAGAGGC |  |
| aceEF-D-2R | TGACTCGCCTTCACCTTCT |  |
| aceEF-W-2F1 | TCAGGAGCTAAGGAAGCTAATGTGTCTGATATGAAGCATGACGT |  |
| aceEF-W-2F2 | TCAGGAGCTAAGGAAGCTAATTTGTCTGATATGAAGCATGACGT |  |
| aceEF-W-2R | CTGGCTCATCCATCTCACC |  |
| P2-F | AAGCTTATTTGAAAAAAGTGTGAC | Amplification of regulatory part P2 |
| P2-R | ATTAGCTTCCTTAGCTCCTGA |  |
| ace1-D-1F | CAATGTGCCGCAACACTAC | Deletion of *ace1* |
| ace1-D-1R | CTCAAGCTCCTTTTGACGCT |  |
| ace1-BamHI-R | CAGTGGATCCCTCAAGCTCCTTTTGACGCT |  |
| ace1-XhoI-F | GACGCTCGAGACAATGGCTCGAAGAGTGC |  |
| ace1-D-2F | AGCGTCAAAAGGAGCTTGAGACAATGGCTCGAAGAGTGC |  |
| ace1-D-2R | TACGGCTGGGCAGGTGAGT |  |
| ace2-D-1F | AACGATGACGGTTCCCTACG | Deletion of *ace2* |
| ace2-D-1R | CGTAGCATCTGCTCTAGCTG |  |
| ace2-BamHI-R | CTGTGGATCCCGTAGCATCTGCTCTAGCTG |  |
| ace2-XhoI-F | GTCGCTCGAGGATTGGCAGAATAAATGAGG |  |
| ace2-D-2F | CAGCTAGAGCAGATGCTACGGATTGGCAGAATAAATGAGG |  |
| ace2-D-2R | GTCAGCAGGTGGCGGTGTT |  |
| ace3-D-1F | GCTTTCCCTTCCGCTTGCT | Deletion of *ace3* |
| ace3-D-1R | AACAATAACCTCAATTTATATCGG |  |
| ace3-BamHI-R | CTGCGGATCCAACAATAACCTCAATTTATATCGG |  |
| ace3-XhoI-F | CTGTCTCGAGTTTCAAACCTTGAAAGCCCCT |  |
| ace3-D-2F | GATATAAATTGAGGTTATTGTTTTTCAAACCTTGAAAGCCCCT |  |
| ace3-D-2R | GTGGCACATATCATTAGCGA |  |
| pflB-D-1F | CCGTCAAATGGTTGGTACTG | Deletion of *pflB* |
| pflB-D-1R | GACATACCTACCTTTTTAGTAGAAAAAAATAC |  |
| pflB-BamHI-R | TCGTGGATCCGACATACCTACCTTTTTAGTAGAAAAAAATAC |  |
| pflB-XhoI-F | GACGCTCGAGGACTGTCGCGAAATAACGTTATAG |  |
| pflB-D-2F | GTATTTTTTTCTACTAAAAAGGTAGGTATGTCGACTGTCGCGAAATAACGTTATAG |  |
| pflB-D-2R | CTATTGCCTAAGTTGGTTCC |  |
| lldh-D-1F | CTATAGCCTTTACCACGAAACG | Deletion of *lldh* |
| lldh-D-1R | CGAGTTTAAAACGGAAACCC |  |
| lldh-BamHI-R | ACGAGGATCCCGAGTTTAAAACGGAAACCC |  |
| lldh-XhoI-F | GGTGCTCGAGTTTTCTCTCCCTGATAATTCTAAAAAATC |  |
| lldh-D-2F | GGGTTTCCGTTTTAAACTCGTTTTCTCTCCCTGATAATTCTAAAAAATC |  |
| lldh-D-2R | CCTAAACGGTAGCCTCTGCAG |  |
| dldh-D-1F | CTCAAACATCTGCCACCTCTAG | Deletion of *dldh* |
| dldh-D-1R | GGTTCTCTCTCGAAATCATTG |  |
| dldh-BamHI-R | TGCTGGATCCGGTTCTCTCTCGAAATCATTG |  |
| dldh-XhoI-F | GGTGCTCGAGATAAGGAATCGAGGAGGATAG |  |
| dldh-D-2F | CAATGATTTCGAGAGAGAACCATAAGGAATCGAGGAGGATAG |  |
| dldh-D-2R | GATGAGAAGAAAGAGCATGTGAAAG |  |
| pps1-D-1F | GCGGTCAAAGTAAGTATCGG | Deletion of *pps1* |
| pps1-D-1R | GTCTTTCTCCAGAAACATTGC |  |
| pps1-BamHI-R | GCTAGGATCCGTCTTTCTCCAGAAACATTGC |  |
| pps1-XhoI-F | GCTCCTCGAGTTCGCGTTAGAATTCTCTAATCT |  |
| pps1-D-2F | GCAATGTTTCTGGAGAAAGACTTCGCGTTAGAATTCTCTAATCT |  |
| pps1-D-2R | GCTTGGTCGTAGAGTGCCT |  |
| pps2-D-1F | TGCGAGAAGGCGGTTTGGT | Deletion of *pps2* |
| pps2-D-1R | AGTCAGCGTCCTTGTTTTGTT |  |
| pps2-BamHI-R | CGTCGGATCCAGTCAGCGTCCTTGTTTTGTT |  |
| pps2-XhoI-F | ATGACTCGAGCGCAAACCTAAACAGCACTATAC |  |
| pps2-D-2F | AACAAAACAAGGACGCTGACTCGCAAACCTAAACAGCACTATAC |  |
| pps2-D-2R | ATGGGCACTGTCACAACGG |  |
| ppc-W-1F | GCCCATTACCTGGCATTAG | Fine-tuning *ppc* gene expression |
| ppc-W-1R | GTCACACTTTTTTCAAATAAGCTTTGTCGTCCTGCCTCGTAAAAAAAC |  |
| ppc-BamHI-R | GTCTGGATCCCGTTCATTGTCGTCCTGCCT |  |
| ppc-XhoI-F | GCTACTCGAGGCAGGTATGCGTAACACTGG |  |
| ppc-W-2F1 | TCAGGAGCTAAGGAAGCTAATATGAACGAGAAATACGCCGC |  |
| ppc-W-2F2 | TCAGGAGCTAAGGAAGCTAATTTGAACGAGAAATACGCCGC |  |
| ppc-W-2R | CTTGCTGGTGGCTCGCTTTC |  |
| 16s-qF | ACCCTTATCCTTGTTTGCC | qRT-PCR analysis of *16S* |
| 16s-qR | CCTCTGTATGCGCCATTGTA |  |
| aceE-qF | TGGCAGTTCCCAACCGTAT | qRT-PCR analysis of *aceE* |
| aceE-qR | ATCCATCTCACCGTCACCC |  |
| ppc-qF | TTGAAACCCTGGACGACTT | qRT-PCR analysis of *ppc* |
| ppc-qR | CATTGCATGGTACTGAGCC |  |
